# Integrated effects of land cover, topography, urban morphology, and PM2.5 on land surface temperature

**DOI:** 10.1371/journal.pone.0344297

**Published:** 2026-05-15

**Authors:** Shibin Ma, Haiquan Xu, Rongfang Xin, Li Huang, Yonghao Hou, Jia Wang, Xuesong Yang

**Affiliations:** 1 Institute of Geological Survey of Qinghai Province, Xining, China; 2 Qinghai-Tibet Plateau During the North Qilian Geology and Mineral Resources Laboratory of Qinghai Province, Xining, China; 3 Qinghai Remote Sensing Big Data Engineering Technology Research Center, Xining, China; The Chinese University of Hong Kong, HONG KONG

## Abstract

Rapid urbanization profoundly alters land surface characteristics, intensifying thermal stress and air pollution, yet the seasonal dynamics of land surface temperature (LST) in high-altitude cities remain poorly understood. This study focuses on Xining, a representative plateau city, to examine how land cover, topography, urban morphology, and atmospheric pollution jointly regulate urban thermal environments. We integrated multi-source remote sensing data into a multidimensional indicator system and applied XGBoost, SHAP, and structural equation modeling to quantify nonlinear contributions, threshold effects, and interaction pathways. The results reveal pronounced spatiotemporal heterogeneity, with the strongest urban heat island effects in summer and dominant topographic regulation in spring and winter. Seasonal shifts in leading drivers were evident: population density was most influential in summer, vegetation indices in autumn, and landscape fragmentation and terrain parameters in spring and winter. Nonlinear thresholds were identified, including enhanced cooling at moderate NDVI values and intensified warming once built-up intensity exceeded critical levels. Significant interaction effects, such as vegetation–impervious coupling in spring and terrain–albedo mediation in winter, further highlighted the complexity of urban thermal regulation. Collectively, these findings advance understanding of seasonally varying mechanisms in plateau cities and provide a scientific basis for climate-resilient urban planning in topographically complex regions.

## 1. Introduction

With rapid urbanization, the share of the global population living in cities is projected to reach ~68% by 2050 [1]. Urban natural land is increasingly replaced by buildings, roads, and other impervious surfaces, altering land use, radiative properties, and surface energy balance, and thereby intensifying multiple environmental risks [2]. Among these, the urban heat island (UHI) and fine particulate pollution (PM2.5) are particularly consequential for ecosystem function and public health [3]. The UHI describes the condition in which urban near-surface or skin temperatures exceed those of surrounding rural areas—a phenomenon documented for decades and widely used as an indicator of urbanization’s thermal imprint [4,5]. In China, heat risk has risen to national prominence given its socioeconomic implications [6,7]. PM2.5 (particles with aerodynamic diameter ≤2.5 μm) is a key indicator of urban air quality due to its well-established cardiopulmonary health burden [8]. The co-occurrence of elevated heat and PM2.5 can compound risks by increasing morbidity and premature mortality, underscoring the need for effective, evidence-based mitigation strategies [9,10].

Urban thermal conditions are typically characterized using air temperature (AT) within the canopy layer and land surface temperature (LST) retrieved by thermal remote sensing [11–13]. AT from meteorological networks provides high temporal resolution and is invaluable for diagnosing canopy-layer processes, but it is sensitive to station siting, network density, and synoptic variability [14,15]. In contrast, satellite-derived LST offers wall-to-wall spatial coverage and intra-urban detail suitable for mapping thermal heterogeneity and diagnosing surface processes [16]. We use LST as a proxy for the urban surface thermal environment. LST has thus become central to studying the spatial patterns and drivers of urban surface thermal environments and to informing place-specific cooling interventions.

A diverse set of environmental factors jointly shapes LST [17]. Land cover composition is a first-order control: higher fractions of impervious surfaces are generally associated with higher LST, whereas vegetation cover exerts consistent cooling via shading and evapotranspiration [18]. Widely used spectral indices capture these contrasts: higher NDVI typically corresponds to lower LST, while higher NDBI, representing built-up intensity, aligns with higher LST. Surface albedo modulates shortwave absorption; high-albedo materials often mitigate peak surface heating, especially in summer [19]. Topography further regulates local energy balance. Steeper slopes may facilitate nocturnal cooling, while slope aspect modulates insolation (e.g., south-facing slopes in the Northern Hemisphere generally receive greater solar input) [19]. Terrain wetness indices (e.g., TWI) capture moisture convergence and soil water availability that can indirectly cool surfaces through enhanced evapotranspiration [20,21]. Urban morphology adds a three-dimensional layer of control. Building height (BH), building density (BD), floor area ratio (FAR), and sky view factor (SVF) co-determine radiative trapping, ventilation potential, and the urban canyon effect [22,23]. Higher BD and FAR often coincide with higher LST due to heat storage and limited longwave escape; lower SVF tends to elevate nighttime LST by restricting radiative cooling; conversely, vegetation density within street canyons can offset warming via transpiration and shading when aerodynamic resistance is not excessive [24]. The relationship between PM2.5 and LST remains less consistently quantified. Aerosol–radiation interactions can both attenuate incoming shortwave radiation (dampening daytime heating) and modify boundary-layer stability (sometimes sustaining higher nighttime temperatures). The sign and magnitude of this modulation are season- and regime-dependent, varying with aerosol optical properties, synoptic conditions, and local emissions.

Despite substantial progress, important gaps remain. Many studies isolate single drivers, overlook nonlinearities and interactions, or underrepresent complex geographic settings such as high-elevation cities [25,26]. High-altitude urban areas are jointly influenced by strong solar radiation, low air density and pressure, dry/cold monsoonal regimes, and complex terrain. These factors can reorder seasonal thresholds and marginal effects of canonical “cooling” (e.g., NDVI, terrain moisture) and “warming” (e.g., NDBI, BD, anthropogenic heat) drivers and can induce seasonally divergent aerosol–temperature coupling [27,28]. Consequently, robust identification of hotspots, cold spots, and “hot-in-summer, cold-in-winter” composite vulnerability often requires a seasonally explicit analysis with attention to multiscale spatial units and three-dimensional urban morphology [29,30]. The Qinghai–Tibet Plateau and cities such as Xining are exemplary settings where warm-season heat events have intensified, nocturnal heat pressure can be high, and terrain-guided ventilation corridors may strongly modulate local energy balance.

Classical statistical approaches (e.g., linear regression, spatial regression) have advanced understanding of LST drivers but can struggle with high-dimensional, multi-scale data and with nonlinear interactions among land cover, topography, morphology, and pollution [23,31,32]. Machine learning has emerged as a complementary toolkit: tree ensembles (e.g., gradient boosting) [33,34], support vector machines [35], and neural networks [36] can flexibly learn complex functional forms from large datasets. Crucially, recent advances in explainable artificial intelligence—notably Shapley Additive Explanations (SHAP) [27,37,38]—enable post-hoc attribution of model predictions to features, quantifying relative importance, nonlinear marginal effects, thresholds, and pairwise interactions [39,40]. This affords a unified way to interrogate multi-driver systems without sacrificing interpretability.

To address the above gaps, we develop an interpretable machine-learning framework to disentangle the integrated effects of land cover, topography, urban morphology, and PM2.5 on LST in Xining, a high-elevation plateau valley city with pronounced terrain heterogeneity. Specifically, we (1) map seasonal LST patterns and quantify their spatial heterogeneity across the central urban area; (2) build season-specific XGBoost models and apply SHAP to identify the relative importance, nonlinear responses, and interaction effects of land cover, terrain metrics (e.g., elevation, slope, and aspect), 3D urban morphology indicators, and PM2.5; and (3) further employ structural equation modeling to test direct and indirect pathways, with particular attention to the potential mediating role of PM2.5 under different seasonal backgrounds. Relative to recent XGBoost–SHAP studies in cities with complex terrain, our novelty lies in a season-resolved, terrain-explicit attribution of dominant drivers and thresholds in a plateau valley setting, combined with mechanism-oriented pathway testing, thereby providing actionable evidence for climate- and health-responsive urban planning in high-altitude environments.

## 2. Materials and methods

### 2.1 Study area

**Fig 1:**
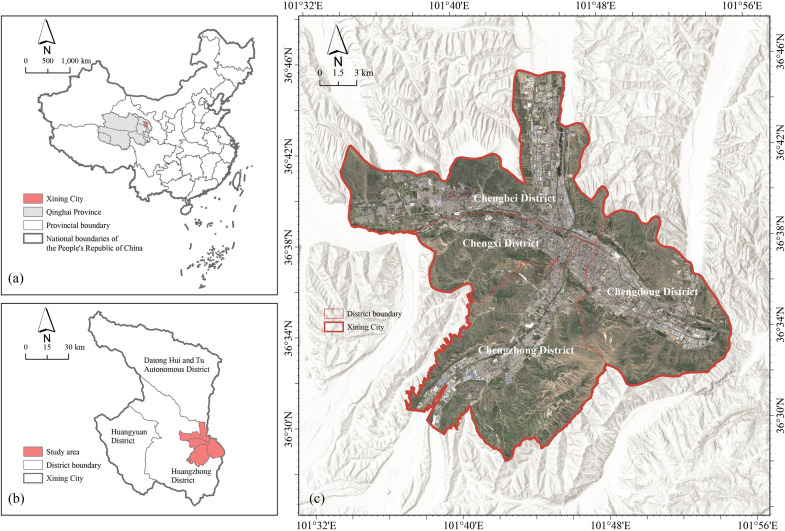
Location of the study area. Administrative boundaries © OpenStreetMap contributors (ODbL). No copyrighted basemap or proprietary map data were used.

This study focuses on the central urban area of Xining, the capital of Qinghai Province in northwestern China. The research domain comprises the four core districts—Chengdong, Chengzhong, Chengxi, and Chengbei (Fig 1)—which together constitute the city’s principal urbanized zone [41]. Geographically, Xining lies on the northeastern margin of the Qinghai–Tibet Plateau within the Huangshui River valley. The city sits at an average elevation of ~2,261 m, with terrain generally sloping from southwest to northeast; surrounding mountains constrain urban expansion within a narrow valley corridor [42].

### 2.2 Data sources and indicators

We integrated multi-source datasets—satellite remote sensing, ground-based meteorological observations, and PM2.5 monitoring—to construct a seasonal indicator system for urban LST and atmospheric pollution. Seasonal LST was derived from Landsat 8 Collection 2 Level-2 imagery, with scenes selected to represent spring, summer, autumn, and winter. PM2.5 concentrations were obtained from the China National Environmental Monitoring Centre. Land cover and vegetation characteristics were derived from the ESA WorldCover dataset, from which landscape metrics (e.g., patch density, largest patch index, aggregation index, edge density, number of patches, cohesion, Shannon diversity index) were computed. Additional predictors included NDVI, NDBI, albedo, and population density (POP). Topographic variables—elevation, slope, aspect, stream power index (SPI), topographic wetness index (TWI), and topographic position index (TPI)—were extracted from the 30-m SRTM DEM. Urban morphology was characterized using Baidu Maps–derived building footprints and attributes to calculate BD, BH, and FAR, complemented by impervious surface proportion and ecological land-use metrics. All datasets were projected to the WGS 1984/ UTM Zone 47N coordinate system to ensure spatial consistency. Within this framework, LST served as the dependent variable, and PM2.5 was introduced as a mediator to capture indirect effects of urban morphology on LST. A concise summary of data sources and processing steps is provided in Table 1.

**Table 1:** Indicators chosen in this study.

Category	Indicators	Abbr.	Description
**Land cover**	Normalized difference vegetation index	NDVI	Measures vegetation cover and health; higher values indicate more lush vegetation.
	Normalized Difference Built-up Index	NDBI	Building coverage index, reflecting the proportion of impervious areas in cities.
	Broadband shortwave surface albedo	Albedo	The ability of the surface to reflect solar radiation; high albedo can reduce LST.
	Impervious surface density	ID	Impervious surface density, reflecting the proportion of hardened surfaces.
	Cropland density	CD	The proportion of herbaceous rain-fed farmland patches represents the level of farmland coverage.
	Vegetation density	VD	Vegetation density, which measures the degree of greening in the vertical direction.
	Number of patches	NP	The number of patches reflects the degree of landscape fragmentation.
	Patch density – needleleaf forest (deciduous/closed)	PD_NF	Closed deciduous needle leaved forest patch density, indicating the spatial distribution density of coniferous forests.
	Aggregation Index – needleleaf forest	AI_NF	Closed deciduous needle leaved forest aggregation index, indicating the connectivity of coniferous forests.
	Patch density – broadleaf forest (deciduous/closed)	PD_BF	The density of closed deciduous broadleaved forest patches represents the spatial distribution of broad-leaved forests.
	Deciduous shrubland density	DSD	The proportion of leaf litter shrub patches reflects the level of shrub coverage.
**Topography**	Slope angle	Slope	The degree of surface inclination affects solar radiation reception and water flow convergence.
	Aspect (slope orientation)	Aspect	Surface orientation, adjusting the angle of solar incidence and heating intensity.
	Topographic Wetness Index	TWI	Topographic Wetness Index, reflecting water accumulation and potential moisture levels.
**Urban** **Morphology**	Building density	BD	Building density, ratio of floor area to total area.
	Building height	BH	Building height affects urban canyon effect and ventilation.
	Floor area ratio	FAR	The floor area ratio is the ratio of the total floor area to the plot area, reflecting the three-dimensional building density.
	Tree height	TH	Tree height reflects the vertical structure of the tree.
**Socioeconomics**	Population density	POP	Population density reflects anthropogenic heat emissions and urban development intensity. persons/km².
**Environment**	Fine particulate matter (≤2.5 μm)	PM2.5	Air pollution indicators can affect radiation flux and LST.

### 2.3 Methods

#### 2.3.1 Research framework.

**Fig 2:**
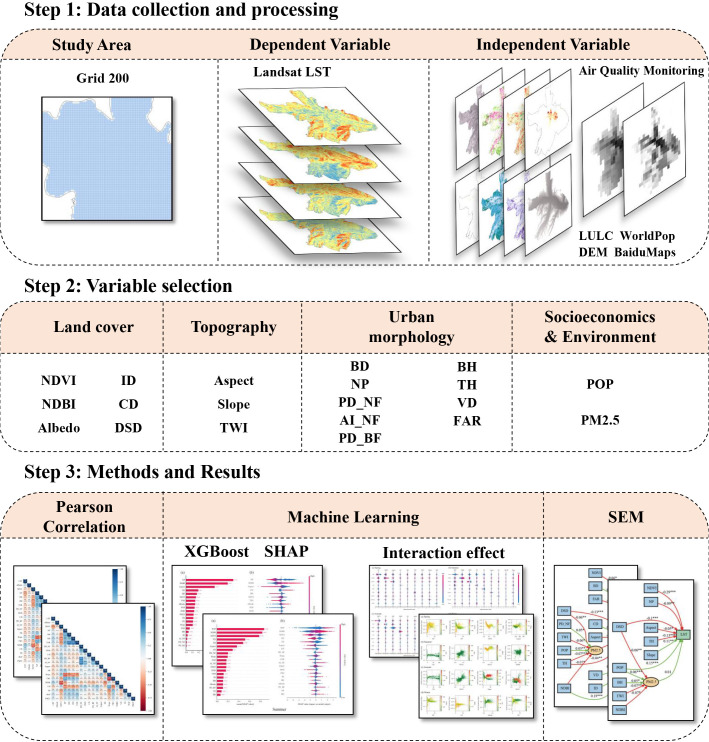
Research framework.

As illustrated in Fig 2, we compiled land cover, topographic, urban morphology, socio economic, and environmental datasets for Xining, China. All layers were reprojected to a common coordinate system and aggregated to the study units via zonal statistics to ensure spatial alignment. After correlation screening, collinearity diagnostics, and feature selection, we retained 20 predictors from an initial set of 85 that best explained LST. We first trained an XGBoost model to derive the global importance ranking of all predictors. We then quantified marginal and pairwise interaction effects using PDP and SHAP. Finally, treating PM2.5 as a mediator, we examined causal pathways from the predictors to LST to disentangle direct and indirect effects.

#### 2.3.2 XGBoost and interpretations.

Classical regression models often struggle to capture the nonlinear and interactive effects among multiple drivers in complex urban settings. We therefore adopt XGBoost [33], a gradient-boosted tree method that is robust and accurate on heterogeneous, high-dimensional datasets. Tree-based learners naturally accommodate nonlinearities and higher-order interactions without feature standardization. Prior to modeling, we conducted pre-screening to minimize redundancy. Pearson correlations indicated strong seasonal covariation among vegetation, built environment, terrain, and LST (S1 Fig). Variables with variance inflation factor > 10 were excluded. The final indicator set is listed in Table 1. The XGBoost model was implemented in Python with LST as the dependent variable and predictors spanning land cover, urban morphology, terrain metrics, and atmospheric/socio-economic attributes. Data were split into training (70%) and testing (30%) subsets. Performance was evaluated using coefficient of determination (R²) and root-mean-square error (RMSE). Hyperparameters were tuned via 10-fold cross-validation to improve generalization and reduce overfitting. The selected values were: max_depth = 6, learning_rate = 0.01, n_estimators = 700, and colsample_bytree = 0.5.

Relative feature importance was assessed using the gain metric (sum of squared error reduction attributable to splits on a feature across all trees). Formally, for feature *x*_*i*_ its overall importance *R*_*xi*_ aggregates the decrease in squared error *△SSE*_*j*_ at each split *j* where *x*_*i*_ is the splitting variable across all trees *T*_*k*_ in the ensemble:


$$\begin{array}{c} R_{x_{i}}=\frac{\text{1}}{m}\sum \overset{m}{\underset{k=\text{1}}{\mathrm{\backslash nolimits}}}\;R_{x_{i}}(T_{k}) \end{array}$$ (1)

$$\begin{array}{c} R_{x_{i}}(T_{k})=\sum \overset{N-\text{1}}{\underset{j=\text{1}}{\mathrm{\backslash nolimits}}}\;\overset{\text{2}}{\underset{j}{D}}I(v_{j}=x_{i}) \end{array}$$ (2)

Where *m* is the number of trees. *R*_*xi*_ (*T*_*k*_) measures how much feature *x*_*i*_ contributes to error reduction in tree *k*; it sums the squared-error decrease at each split, counting only the nodes where *x*_*i*_ is the splitting feature. To enhance interpretability, we use SHAP [43], which decomposes each prediction into additive feature contributions grounded in cooperative game theory [44]. The Shapley value for feature *i* is


$$\begin{array}{c} \phi_{i}(f)=\sum {\mathrm{\backslash nolimits}}_{S\subseteq N\{i\}}\;\frac{|S|!\cdot \left(\right|N\left|-\right|S|-1)!}{|N|!}\cdot [f(S\cup \{i\})-f(s)] \end{array}$$ (3)

Where *N* is the set of all features, *S* is a subset excluding *i*, and *f*(*S*) denotes the model’s expected prediction conditional on the included features. The model enables us to analyze the significance of variables and gain insights into the precise impact of each feature on the model’s output. For the nonlinear response and synergistic effects between key driving factors, and interaction plots are used for visualization.

#### 2.3.3 Structural equation modeling.

To test the hypothesized mediating role of PM2.5 in the driving factors–LST relationship, we employ structural equation modeling (SEM) [45] to quantify the indirect effect of PM2.5. SEM is well suited to disentangling causal pathways by jointly estimating direct and indirect effects, incorporating latent constructs, and accounting for measurement error [46].

Guided by prior applications in urban climate studies, we specified a seasonal path model in which UM indicators (e.g., BD, BH, NDVI, POP, topographic indices) are exogenous variables, LST is the outcome, and PM2.5 is the mediator. Predictor selection was informed by XGBoost feature importance and Pearson correlations to retain variables strongly associated with LST. The model was implemented in R (lavaan) with maximum likelihood estimation. To maintain parsimony, only statistically significant paths (p < 0.05) were retained. Separate SEMs were fitted for each season. Goodness of fit was assessed using the goodness-of-fit index (GFI) and standardized root-mean-square residual (SRMR); higher R² values indicate stronger explanatory power [47]. As reference thresholds, GFI > 0.90 typically indicates good fit (GFI > 0.85 acceptable), and SRMR < 0.08 suggests good fit.

## 3. Results

### 3.1 Spatio-temporal patterns of LST

**Fig 3:**
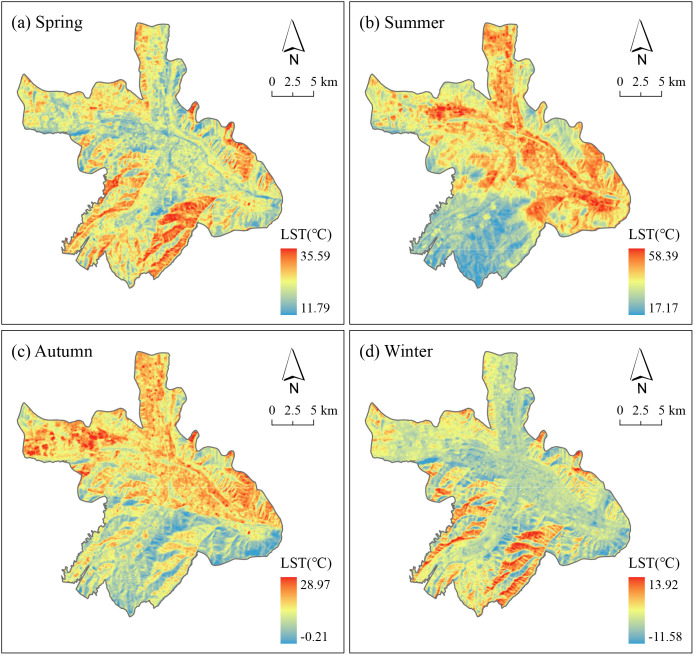
Seasonal maps of LST (°C) for Xining’s central urban area: (a) spring, (b) summer, (c) autumn, and (d) winter. Warmer colors denote higher LST. Color scales differ by panel to reflect seasonal ranges; scale bar and north arrow shown. Administrative boundaries © OpenStreetMap contributors (ODbL). No copyrighted basemap or proprietary map data were used.

The spatial distribution of LST in Xining, derived from Landsat imagery (Fig 3), shows pronounced seasonal variability superimposed on a persistent urban–rural LST gradient. LST follows a clear annual cycle, peaking in summer (17.2–58.4 °C) when a strong UHI intensifies over the urban core and densely built districts, and declining sharply in winter (down to −11.6 °C) when high-elevation and vegetated zones act as distinct cold islands. Transitional conditions in spring (11.8–35.6 °C) and autumn (−0.2–29.0 °C) yield intermediate LST states. Spatially, hotspots remain anchored in the urban core year-round, whereas cooler areas consistently occur along the southwestern mountainous periphery and within vegetation-rich patches. In summer, LST hotspots extend northward and eastward, reflecting the coupling of recent urban expansion with heat accumulation; in winter, the northern sector—exposed to prevailing northwesterly winds—exhibits enhanced cooling. Topographic complexity further accentuates heterogeneity: slope–aspect interactions modulate local energy balance, with southeast-facing slopes receiving prolonged insolation and showing elevated LST, while shaded northwest-facing slopes remain cooler, consistent with aspect–radiation theory. Seasonal inversions also appear in spring and winter, when some built-up areas register lower LST than adjacent bare mountain slopes. This pattern likely reflects a combination of factors, including low solar elevation that amplifies radiative gains on sun-facing slopes, Landsat’s morning overpass capturing urban surfaces with high LST inertia still relatively cool, and transient snowmelt or exposed soils increasing slope–urban contrasts. Collectively, these results indicate that Xining’s LST is jointly governed by seasonal UHI dynamics, topographic modulation, vegetation distribution, and spatial heterogeneity in building density. Overall, the cross-season persistence of slope–aspect contrasts and valley–mountain differentials (Fig 3), together with their stronger expression in cooler seasons, indicates that topography continuously regulates local radiative loading and heat storage, acting as a year-round constraint on the urban thermal environment rather than a static background factor.

### 3.2 Relative impacts of driver factors on LST

**Fig 4:**
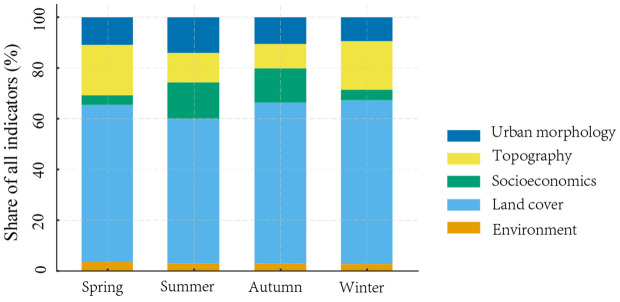
Seasonal composition of all indicators by category; each seasonal bar sums to 100%, highlighting the dominance of land cover and the seasonal shifts in topography and urban morphology.

SHAP analyses of the XGBoost model—robust across seasons (all-season R² > 0.60)—show that LST is governed by a seasonally shifting triad of drivers (Fig 4): vegetation–terrain cooling, anthropogenic intensity, and built-up/radiative retention (Fig 4). Across seasons, land cover is the dominant contributor to the indicator mix, accounting for 62.0% (Spring), 57.0% (Summer), 63.5% (Autumn), and 64.5% (Winter). Topography forms a consistent second tier that strengthens in the cold seasons—19.8% (Spring) and 19.1% (Winter) versus 11.6% (Summer) and 9.7% (Autumn)—indicating enhanced terrain control when LST is weaker. The strengthened topographic contribution in spring and winter suggests that terrain-related regulation persists across seasons and becomes more dominant when background LST is lower and biophysical cooling is constrained. Urban morphology remains moderate overall (10.9%, 14.1%, 10.5%, 9.4% from Spring to Winter), with a summer uptick aligned with taller canopies/buildings and density effects. Socioeconomics (driven by population density) surges in Summer/Autumn (14.4%/13.5%) but is marginal in Spring/Winter (3.8%/4.2%), reflecting seasonal intensification of human activity. Environment (PM2.5) contributes the least and is relatively stable (~2.8–3.5%). Overall, the composition highlights land cover as the structural baseline, topography as a seasonally amplified control in cooler periods, and morphology–human factors as summer-skewed modifiers.

**Fig 5:**
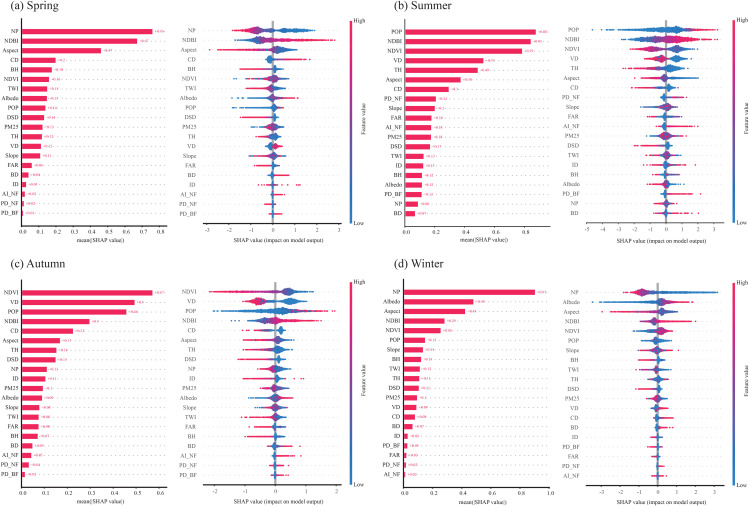
Seasonal ranking of global SHAP importance (left; mean |SHAP|) and local effect distributions (right; beeswarm) for the key predictors. Panels (a–d) correspond to spring, summer, autumn, and winter. Positive SHAP values indicate warming effects on LST; negative values indicate cooling. Color encodes feature value (red = high, blue = low).

Interpreting SHAP (bars = global importance; beeswarm = signed local effects; positive = warming, negative = cooling), we find that vegetation structure and greenness (NDVI, VD, TH), together with terrain factors, constitute the primary cooling complex in the transitional seasons, while their leverage contracts in winter due to dormancy (Fig 5). Anthropogenic intensity—proxied by POP and NDBI—is the dominant warming complex in summer, with wider SHAP dispersion indicating stronger spatial heterogeneity. In winter, NDBI and Albedo, assisted by planform configuration (NP, Aspect), become the principal retention complex, sustaining higher LST despite weaker biophysical cooling. Across seasons, two robust patterns emerge. First, directionality is stable: vegetation/terrain → negative SHAP (cooling); population/built-up → positive SHAP (warming). Second, several drivers are threshold-sensitive: warming accelerates in the upper quantiles of POP/NDBI, whereas NDVI/VD exhibit stronger cooling beyond mid-range levels—implying diminishing returns at low vegetation and steeper benefits once structural canopy is established. Overall, vegetation–terrain cooling ranks first in transitional periods, anthropogenic intensity leads in summer, and built-up/radiative properties lead in winter. These results motivate season-specific mitigation: prioritize canopy continuity/volume and high-Albedo retrofits for hot months, maintain vegetation structure to exploit spring–autumn leverage, and manage impervious configuration and canyoning where winter retention is strongest.

### 3.3 Marginal and interaction effects of driver factors on LST

**Fig 6:**
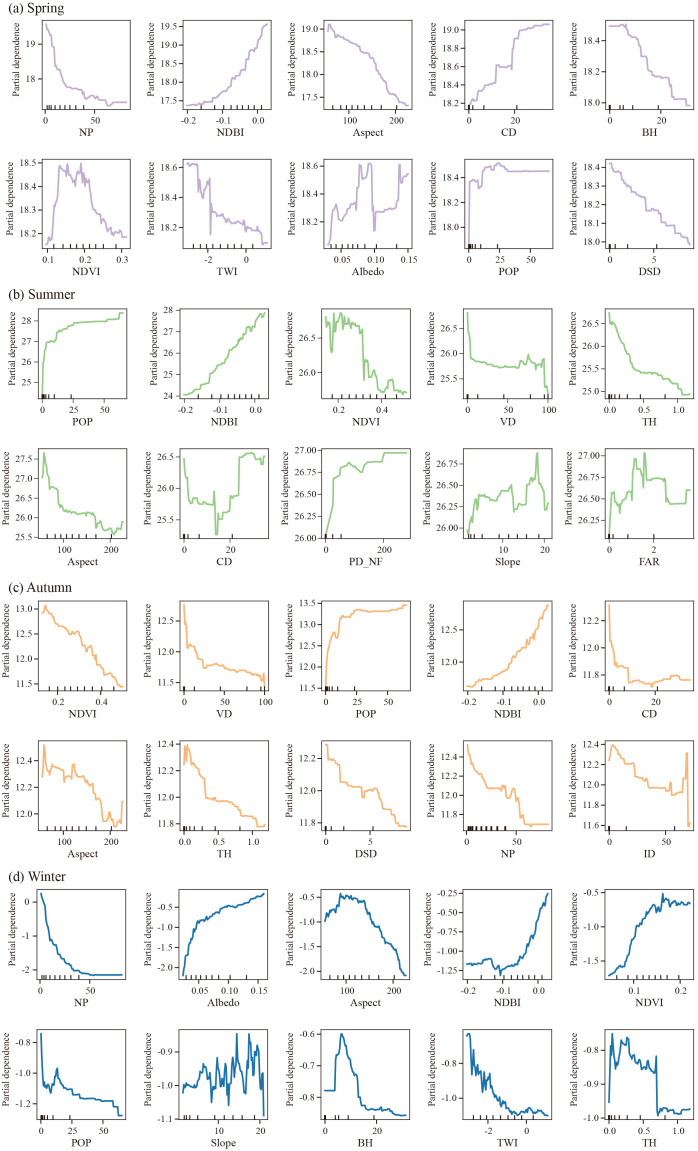
Seasonal PDP curves for the plotted driver factors. Curves show each factor’s marginal effect on LST (ticks denote the empirical distribution of factor values).

Seasonal PDPs show that LST responds to driver factors in nonlinear, threshold-dependent ways (Fig 6). Vegetation metrics (NDVI, VD, TH) provide robust cooling with diminishing returns beyond seasonal breakpoints: cooling strengthens around NDVI ≈ 0.2–0.3 in spring–autumn and stabilizes after ≈ 0.4 in summer; in winter, weak greenness can correspond to slight warming, consistent with vegetation dormancy. Topography/structure exhibits multi-stage effects: Slope and TH yield stronger cooling once past moderate levels; Aspect shows enhanced mitigation near 150°–200°; TWI and BH display two-phase responses reflecting moisture and canyoning controls. Anthropogenic and built-up proxies are persistent warming forces: NDBI increases LST almost monotonically with the steepest rise around −0.1 to 0.1; POP shows a clear threshold near ≈ 25 persons/km², above which LST remains elevated with a gentler slope; Impervious surface density (ID), cropland density (CD) and FAR also exhibit positive associations where they appear. Landscape configuration tends to moderate heat: NP contributes additional cooling once > ~40 patches in spring–winter, while DSD above ~5% enhances mitigation in autumn. Albedo plays a seasonal radiative role— < 0.05 amplifies winter warming but higher values attenuate it; PD_NF shows weak-to-moderate warming in summer at higher patch densities, and slope has mild U-shaped behavior in some seasons.

To summarize the nonlinearities shown in Fig 6, PDP/SHAP diagnostics suggest three key breakpoints associated with disproportionate LST responses: NDVI ≈ 0.2–0.3 (cooling strengthens), NDBI ≈ −0.1 to 0.1 (steepest warming), and POP ≈ 25 persons/km² (warming inflection), with season-specific saturation beyond these ranges.

Overall, SHAP results: vegetation/topography deliver nonlinear, threshold-governed cooling, whereas NDBI/POP (and where present, ID/FAR/CD) sustain warming. These shapes identify actionable levers—e.g., raising NDVI into effective ranges, increasing canopy structure (VD/TH), and keeping NDBI/POP near or below identified breakpoints—to implement season-specific, indicator-targeted heat-mitigation strategies.

**Fig 7:**
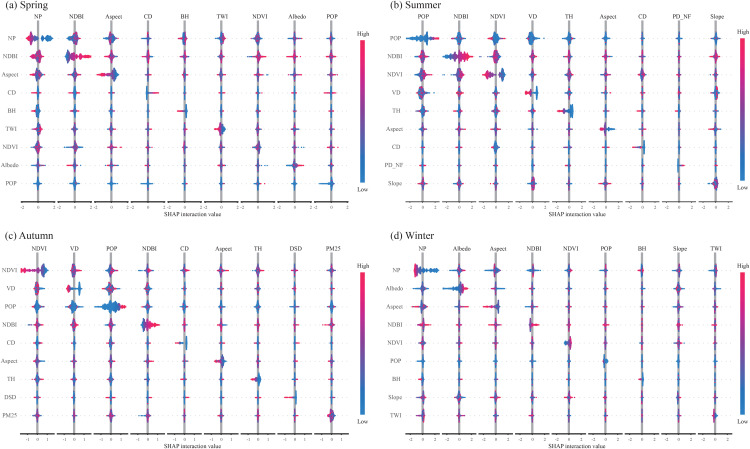
Seasonal SHAP interaction beeswarms for the top driver factors (columns = source factor; positive = synergistic warming, negative = joint cooling).

SHAP interaction values indicate three robust, complementary mechanisms governing joint effects on LST (Fig 7). First, vegetation structure (NDVI/VD/TH) systematically moderates the warming induced by anthropogenic and built-up intensity (POP/NDBI): medium–high vegetation levels offset these pressures, whereas sparse vegetation synergizes with them to amplify heat. Second, radiative–planform coupling (Albedo × NP/Aspect) regulates LST: higher Albedo combined with more dispersed planforms or favorable orientations reduces heat storage, while low Albedo with compact/unfavorable orientations enhances it. Third, topographic–ventilation controls (Slope/TWI/BH interacting with NP/Aspect) provide secondary, generally offsetting modulation consistent with airflow and insolation pathways. Collectively, these patterns prioritize co-design levers: increase canopy volume/continuity where POP/NDBI are high, pair Albedo retrofits with planform/orientation optimization in retention hotspots, and exploit ventilation corridors to fine-tune local cooling.

**Fig 8:**
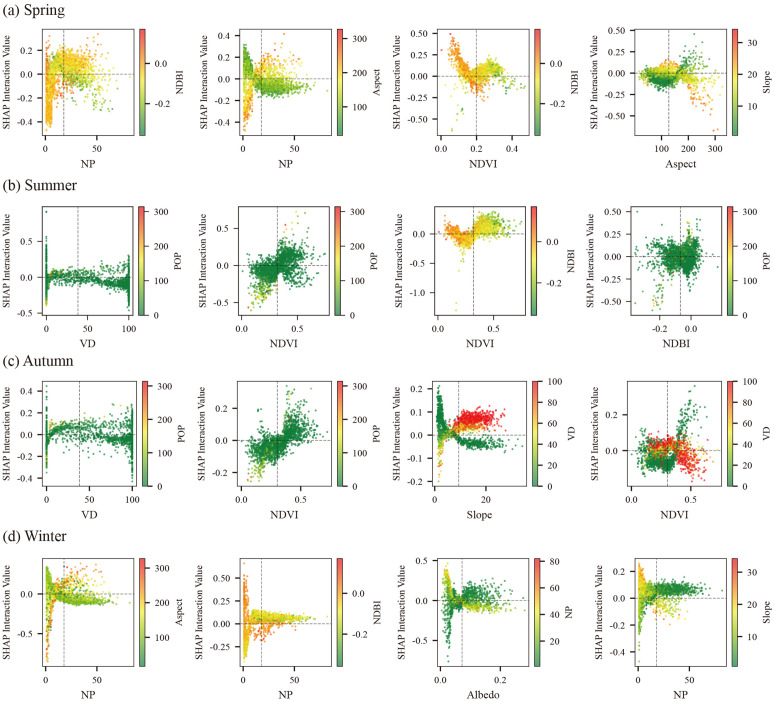
Seasonal bivariate SHAP interactions among key LST drivers.

Fig 8 reveals that LST is shaped by a few robust, seasonally shifting interaction logics rather than isolated predictors. Vegetation (NDVI/VD) delivers the strongest cooling, but only under low–moderate urbanization: its effect saturates and is partly cancelled where NDBI/POP are high, indicating clear thresholds (NDVI≈0.25–0.35; VD ≈ 40–50%). Urban morphology acts as a double-edged sword: higher patchiness (NP) cools slightly on less-impervious surfaces and favorable terrain, but turns warming when paired with high NDBI—a pattern most pronounced in winter. Terrain modulates everything: steeper slopes amplify vegetation cooling and blunt NDBI/NP warming via ventilation and runoff; Aspect strengthens warming on sun-facing slopes and enhances cooling on shaded slopes. Seasonal phase matters—summer–autumn are biophysical (evapotranspiration) dominated, whereas winter shifts to radiative/structural controls (Albedo, Aspect) with NP×NDBI warming unless Albedo is high. Practically, heat-mitigation must co-optimize levers: pair greening with imperviousness control and POP management, exploit slope/Aspect (ventilation, shading), and in winter raise Albedo and limit fragmentation in sun-exposed, flat, heavily built areas.

### 3.4 Seasonal mediation effects of PM2.5 on LST

**Fig 9:**
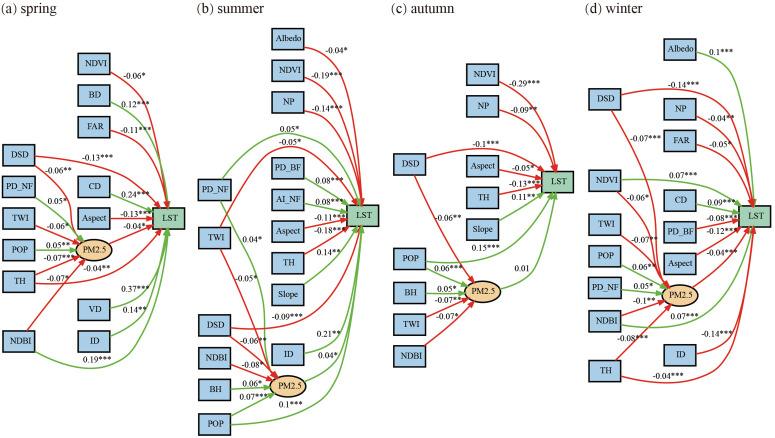
Seasonal SEM paths linking urban morphology/landscape, PM2.5, and LST. Panels (a–d) correspond to spring, summer, autumn, and winter, respectively. Values are standardized path coefficients; asterisks denote statistical significance (p < 0.05, p < 0.01, p < 0.001). Paths without asterisks are not significant; notably, the PM2.5 → LST path is non-significant in autumn (≈ 0.01) but positive in summer (≈ + 0.04).

Building on this, the mediation role of PM2.5 can be made explicit: the pollution pathway carries a small but systematic share of the total effect (standardized paths ≈ |0.04|), comparable to several direct coefficients, and its sign reverses with season (Fig 9). Notably, PM2.5 mediates LST in summer (positive indirect effect, ≈ +0.04), whereas in autumn the indirect (mediated) pathway is not significant, indicating a seasonal weakening/breakdown of the pollution transmission mechanism from upstream drivers to LST during the transition season. In spring/winter, higher PM2.5 dampens LST (≈ –0.04) via aerosol scattering/short-wave dimming under stable boundary layers, thereby amplifying the net cooling contributed by favorable terrain (Aspect/Slope) and greenness. In summer, PM2.5 reinforces warming (≈ + 0.04) as elevated loads co-vary with anthropogenic heat, weak dispersion, and strong insolation, partly offsetting vegetation’s direct cooling. Although autumn shows no significant mediation, upstream factors that shape pollution—e.g., POP, ID, TH, TWI, Slope and Aspect—still regulate the potential for mediation by altering emissions, ventilation, and deposition. Taken together, PM2.5 functions as a season-contingent conduit through which morphology and terrain propagate to LST, and its contribution—while modest—shifts the balance between warming and cooling processes enough to matter for heat-risk management in plateau cities.

## 4. Discussion

### 4.1 Key drivers and seasonal regulation of LST

In this high-elevation city, LST is governed by seasonally reweighted synergies among vegetation (NDVI, VD), imperviousness (NDBI), population pressure (POP), and terrain (Aspect, Slope, TWI), further modulated by solar radiation and spatial variability in surface heat capacity. This overall pattern is consistent with multi-city analyses showing that vegetation loss, impervious expansion and socio-economic intensity are first-order drivers of UHI across diverse climatic and developmental contexts [48,49].

Interpretation of SHAP importance shows that NDBI, Aspect, NDVI, and POP remain persistently influential across seasons, confirming the durable roles of surface radiative properties, terrain-controlled insolation, and human activity. Similar negative NDVI–LST and positive NDBI–LST relationships have been widely reported, including in European and Asian cities where Landsat-based analyses demonstrated robust cooling associated with higher NDVI and warming associated with higher NDBI [50,51]. Our results extend these findings by identifying an optimal NDVI cooling band around 0.2–0.3 and heightened NDBI sensitivity near zero, implying that in plateau valley cities small changes in greenness or imperviousness near these “critical states” can trigger disproportionately large thermal responses.

Landscape fragmentation (NP) shows stronger cooling contributions in spring and winter in Xining, whereas vegetation-related cooling peaks in summer and autumn, indicating a clear seasonal reweighting between configuration and composition effects. This pattern is consistent with landscape-ecological evidence that configuration metrics (e.g., patch number, connectivity, and edge density) can be as important as area in shaping UHI patterns [52]. Importantly, the direction of the configuration effect appears geomorphology-dependent: while studies in lowland megacities often report that excessive fragmentation erodes cooling by breaking up large vegetated cores and increasing edge exposure to impervious heating, in plateau valley cities moderate fragmentation aligned with valley floors and corridors may instead facilitate terrain-guided ventilation and cold-air drainage. Consistent with this mechanism, we observe additional cooling once NP exceeds ~40 patches in spring–winter, suggesting that configuration–LST relationships are not directly transferable from flat cities to high-relief basins without adjustment.

In summer, POP interacts strongly with built-up intensity to amplify warming: LST rises sharply once population exceeds ~25 persons per unit area, a threshold coherent with anthropogenic-heat theory that links traffic flows, HVAC use and metabolic heat to SUHI strengthening [53]. At the same time, topography acts as a year-round regulator of the urban thermal environment in Xining through coupled radiation–moisture–ventilation pathways. Specifically, (1) aspect controls insolation asymmetry (e.g., southeast-facing slopes are persistently warmer than shaded northwest-facing slopes), (2) slope and valley geometry regulate ventilation and cold-air drainage that modulate heat storage and mixing—especially in cooler seasons, and (3) terrain moisture convergence, captured by TWI, maintains valley wetlands as stable cool sources via moisture retention and drainage-related cooling [54]. The magnitude of these terrain effects is amplified by steep relief and strong diurnal radiative forcing, reinforcing the need to treat topography as an active planning constraint rather than a static background.

PM2.5 functions both as mediator and direct contributor. Its positive association with LST in summer is consistent with aerosol–radiation forcing that suppresses nocturnal long-wave cooling and modifies short-wave scattering, as documented for Beijing and Nanjing where fine particles can weaken or reverse canonical UHI patterns depending on season and boundary-layer structure [55, 56]. In autumn, the PM2.5 effect notably attenuates as vegetation and terrain regain more direct control over energy partitioning, aligning with recent evidence that aerosol impacts on SUHI are circulation-regulated and can diminish when synoptic ventilation improves. Taken together, these patterns align with multi-city UHI literature that reports robust vegetation cooling, impervious warming, and persistent terrain control, while extending that evidence to plateau contexts by quantifying actionable thresholds—such as the NDVI band around 0.2–0.3, heightened NDBI sensitivity near zero, and a POP threshold near 25 persons per unit area—that can guide season-specific mitigation in high-elevation valley cities [57]. The disappearance of the PM2.5-mediated pathway in autumn likely reflects transitional atmospheric conditions (e.g., changing boundary-layer structure and dispersion), under which PM2.5 covariation with surface heating becomes less stable than in summer.

### 4.2 Cross-variable interactions and actionable insights

SHAP interaction maps indicate that LST in this plateau city is regulated less by isolated nonlinearities than by seasonally shifting cross-variable synergies among vegetation, imperviousness, population and terrain. Transitional seasons (spring and autumn) exhibit the densest interaction networks, in which landscape configuration, surface properties and topography jointly enhance cooling. In extreme seasons, the network reconfigures: human-activity × impervious coupling dominates in summer, whereas terrain–radiation coupling dominates in winter. This dynamic architecture is broadly consistent with process-based and empirical work suggesting that the strength and sign of UHI drivers are seasonally modulated by background circulation, atmospheric stability and surface energy partitioning [58–60].

A key finding is the strong vegetation × built-up synergy in the warm half of the year. In spring and summer, moderate greenness embedded within impervious contexts sharply lowers LST, whereas additional greening yields diminishing returns in already green areas. Similar context dependence has been reported in sub-pixel mixture and patch-scale studies, where the largest marginal cooling benefits occur when trees and shrubs are strategically integrated into built fabrics rather than concentrated in isolated parks [61,62]. Mechanistically, canopy shading over high-heat-capacity pavements, increased aerodynamic roughness that promotes convective removal, and localized advection from cooler vegetated patches to adjacent impervious surfaces together explain this synergy. Our results add to this literature by identifying explicit NDVI–NDBI combinations at which these processes become most effective, offering clear design targets for mixed-use blocks in plateau cities. The vegetation × population moderation—lower LST under moderate POP where adequate canopy is maintained—echoes multi-city findings that tree-cooling efficiency depends on behavioral intensity and maintenance or irrigation regimes that sustain canopy vigor in densely used spaces [63,64]. In relatively low-density areas, additional canopy may be underutilized or poorly maintained, whereas in very dense cores biophysical cooling can be offset by anthropogenic heat and soil sealing. Here, the interaction surfaces suggest that maintaining intermediate densities coupled with sufficient tree cover can flatten the otherwise steep POP–LST gradient observed above the ~ 25-person threshold, highlighting an important lever for demand-side thermal management. In autumn, strengthened vegetation × terrain coupling is consistent with ecohydrological controls: Slope and Aspect govern light and soil moisture, modulating how much of vegetation’s potential cooling is realized as transpiration declines. This aligns with studies in hilly regions where the cooling effect of vegetation is strongest on aspects with favorable radiation–moisture combinations and weakens on dry, sun-exposed slopes [65]. In winter, Aspect × Albedo interactions regulate radiation exposure and surface energy balance on sun-facing slopes; high-Albedo surfaces can partially offset Aspect-driven warming, whereas low-Albedo materials exacerbate it. Such Aspect–Albedo controls have been observed in mountainous and semi-arid cities, but our findings show that the combination of thin atmosphere and steep relief in plateau basins further magnifies these interactions. PM2.5-associated interactions reinforce its role as both mediator and modifier of cross-variable synergies. Elevated PM2.5 concentrations amplify summer LST in densely built valley floors, where aerosol-induced reductions in long-wave cooling and stabilization of boundary layers disproportionately affect areas already constrained by weak ventilation; in contrast, similar concentrations over wind-exposed slopes exert smaller or even slightly cooling effects, consistent with recent work on spatially heterogeneous aerosol–LST coupling [66–68]. The resulting pattern is that morphology–pollution–LST interactions are strongest in topographically trapped basins, implying that emission control in these “thermal–pollution hotspots” can yield co-benefits for both air quality and thermal comfort.

Translationally, these interaction architectures map onto season-specific interventions. In hot months, priority should be given to integrating trees and other vegetation into impervious fabrics in high-density grids, while simultaneously managing anthropogenic heat through building energy efficiency and transport demand management. In shoulder seasons, planning strategies should emphasize permeability, connectivity and Slope–vegetation coupling—protecting and enhancing green–blue corridors along valley floors and gentle slopes to maximize cold-air drainage. In winter, optimizing surface materials and Albedo on sun-exposed slopes, while avoiding excessive sealing in concave topographic units, can moderate Aspect-induced heating without compromising snowmelt or safety. The identified thresholds—NDVI ≈ 0.2–0.3, NDBI sensitivity near zero, and POP ≈ 25 persons/km²—thus provide quantitative waypoints to operationalize these designs in plateau cities where topography, morphology and pollution are tightly intertwined.

### 4.3 Limitations and future work

Several limitations should be considered when interpreting these findings. First, the analysis is based on seasonal satellite snapshots and gridded PM2.5 products, which inevitably underrepresent short-lived heat extremes, pollution episodes, and intra-seasonal variability. This temporal sparsity may bias our understanding toward “typical” conditions and underestimate the role of transient weather regimes or extreme events. Second, focusing on a single high-elevation valley city (Xining) enhances relevance for plateau contexts but constrains generalizability. The thresholds and interaction patterns identified here may shift under different background climates, urban morphology, and development trajectories.

Future work should therefore integrate multi-year, multi-season and multi-city datasets to better capture temporal variability and spatial heterogeneity, particularly across the broader “third pole” region and other mountainous urban systems. Coupling remote-sensing products with physically based urban climate and ecohydrological models would allow mechanistic testing of the pathways inferred from SHAP analysis, including the joint roles of terrain, vegetation, pollution and anthropogenic heat. Assimilating dynamic meteorology—such as boundary-layer structure, synoptic circulation and extreme weather events—would further clarify weather–morphology–pollution co-variability and improve the attribution of LST responses. Methodologically, extending explainable machine-learning diagnostics with causal inference frameworks and longer time series (e.g., panel data, quasi-experimental designs) offers a promising route to strengthen causal claims and enhance the policy transferability of results for complex-terrain cities.

## 5. Conclusion

This study demonstrates that LST heterogeneity in a high-altitude valley city emerges from seasonally reweighted, terrain-conditioned synergies among vegetation, imperviousness, population, and PM2.5, with topography acting as a persistent regulator of radiative exposure, moisture pathways and cold-air drainage. Rather than being driven by any single factor, the urban thermal environment reflects shifting interaction architectures in which human activity, land cover and terrain exchange dominance across seasons.

By combining XGBoost with SHAP, we quantify both marginal and interaction effects and identify robust, actionable thresholds: an optimal NDVI cooling band near 0.2–0.3, heightened NDBI sensitivity around zero, and a POP inflection at ~25 persons/km². These thresholds provide concrete targets for climate-sensitive design in plateau cities. The interaction structures translate directly into seasonally adaptive strategies: integrating trees and other vegetation into impervious fabrics and managing density and anthropogenic heat in summer; prioritizing permeability, connectivity and Slope–vegetation coupling in spring and autumn to maximize ventilation and cold-air drainage; and optimizing Albedo and surface materials on sun-facing slopes in winter to moderate Aspect-driven warming.

Conceptually, the framework advances UHI theory by showing how complex topography reweights canonical drivers and amplifies cross-variable synergies, particularly those involving terrain and pollution. Practically, it offers a transferable, data-driven “playbook” for plateau and other complex-terrain cities, in which explainable machine learning is used not only to predict LST but to reveal leverage points for season-specific mitigation. Scaling the approach to longer time series, dynamic meteorology and coordinated multi-city comparisons will be essential for refining these thresholds and strategies under ongoing urbanization and climate change.

## Supporting information

S1 Fig Pearson correlation heat map. (ZIP)
