# Supplementary figures and images for: Integrated effects of land cover, topography, urban morphology, and PM2.5 on land surface temperature
Source: PLoS One. 2026 May 15;21(5):e0344297. doi: 10.1371/journal.pone.0344297 (PMC13178901; doi:10.1371/journal.pone.0344297)

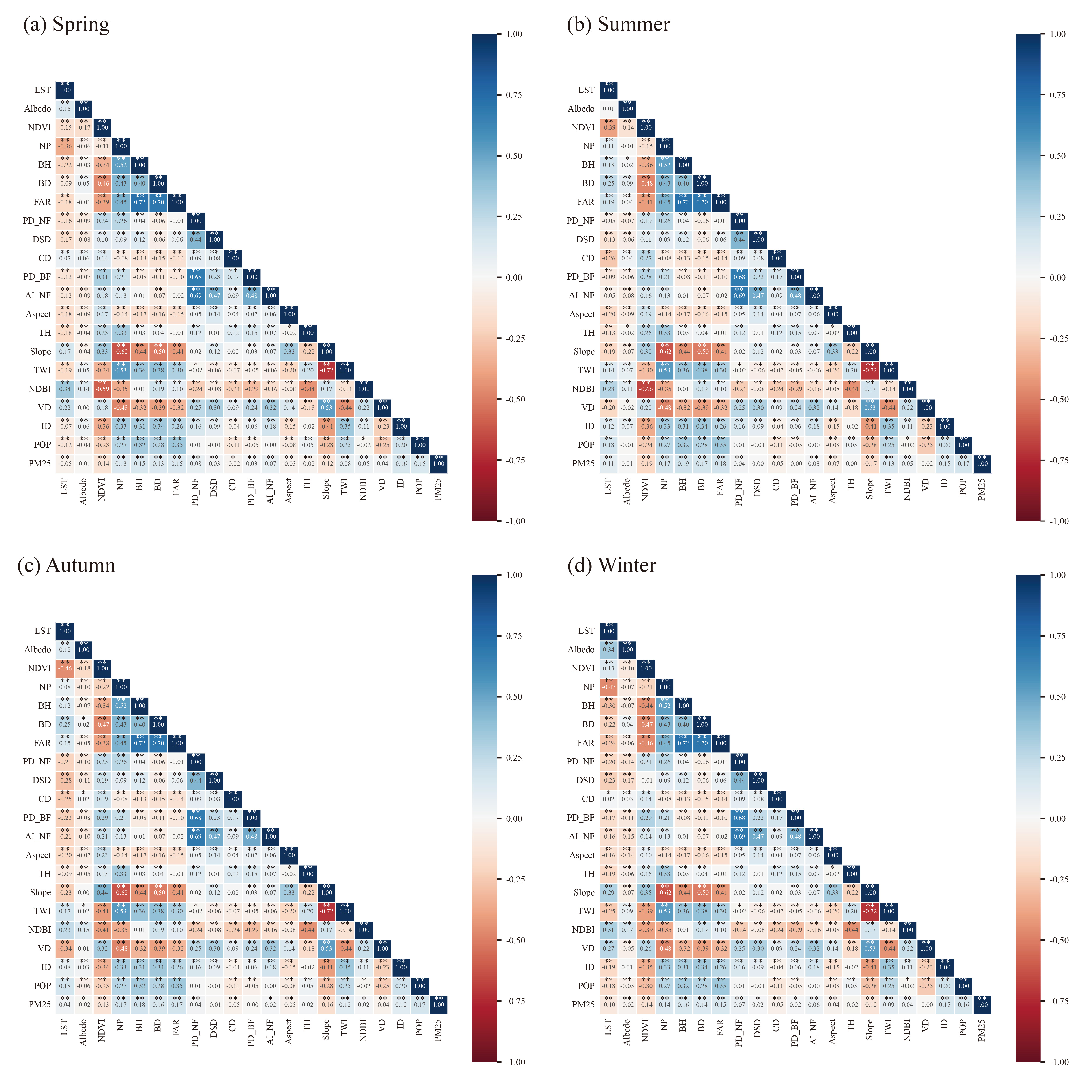

Supplement: S1 Fig — (ZIP) [file pone.0344297.s001.zip › S1 FIG/S1 FIG.jpg]
